# Is Anticoagulation Necessary for Severely Disabled Cardioembolic Stroke Survivors?

**DOI:** 10.3390/medicina55090586

**Published:** 2019-09-13

**Authors:** Kristaps Jurjans, Baiba Vikmane, Janis Vetra, Evija Miglane, Oskars Kalejs, Zanda Priede, Andrejs Millers

**Affiliations:** 1 Department of Neurology and Neurosurgery, Riga Stradins University, 16 Dzirciema Street, LV-1007 Riga, Latvia; evija.miglane@stradini.lv (E.M.); Zanda.priede@stradini.lv (Z.P.); andrejs.millers@stradini.lv (A.M.); 2 Department of Doctoral Studies, Riga Stradins University, 16 Dzirciema Street, LV-1007 Riga, Latvia; 3 Department of Neurology, Pauls Stradins Clinical University Hospital, 13 Pilsonu Street, LV-1002 Riga, Latvia; bvikmane@inbox.lv (B.V.); vetra.janis@gmail.com (J.V.); 4 Faculty of Continuing Education, Riga Stradins University, 16 Dzirciema Street, LV-1007 Riga, Latvia; 5 Department of Internal Medicine, Riga Stradins University, 16 Dzirciema Street, LV-1007 Riga, Latvia; okalejs@gmail.com; 6 Latvian Centre of Cardiology, Pauls Stradins Clinical University Hospital, 13 Pilsonu Street, LV-1002 Riga, Latvia

**Keywords:** atrial fibrillation, cardioembolic stroke, stroke functional outcome, stroke mortality

## Abstract

*Background and Objectives:* Oral anticoagulants are the hallmark of cardioembolic stroke prevention, but they are frequently underused, especially in elderly patients and patients with paroxysmal atrial fibrillation. In our paper, we analyzed the long-term outcome of severely disabled cardioembolic stroke survivors depending on the prescribed antithrombotic secondary prevention medication. *Materials and Methods:* In our study, we retrospectively collected data for ischemic stroke (IS) patients treated in P. Stradins Clinical University hospital, Riga, Latvia, from 2014 until 2017. Patients’ clinical data were collected using local stroke registry, including patients’ demographic data, vascular risk factors, clinical findings, and laboratory results. Severely disabled stroke survivors were followed up by phone at 30/90/180/365 days after discharge. Patients’ functional outcomes were assessed using the adapted version of The Rankin Focused Assessment–Ambulation. The collected data were compared in 4 groups according to prescribed secondary prevention medication. *Results:* A total of 682 (91.42%) patients were followed up and included in data analysis. The median age of patients was 80 (IQR = 75–85) years. Of these patients, 231 (31%) were males and 515 (69%) were females. One-year probability of survival of patients not taking any preventive medication was 53% (IQR = 29–76), while in patients taking antiplatelet agents it was 57% (IQR = 37–78), 78% (IQR = 68–88) of patients on Vitamin K antagonists (VKA) and 81% (IQR = 72–90) in patients on direct oral anticoagulants (DOACs). One year after discharge 73 (31%) had mRS 0–2, 50 (20.9%), 29 (12.1%) were still severely disabled, and 87 (36.4%) had died. *Conclusions:* Anticoagulant use in secondary prevention predicts better functional outcome and higher survival rate in patients with severe cardioembolic stroke due to non-valvular atrial fibrillation (NVAF), therefore severe neurological deficit must not be a reason of restriction of anticoagulation.

## 1. Introduction

Cardioembolic stroke is one of the main ischemic stroke (IS) subtypes along with atherothrombotic stroke and stroke due to small vessel disease [1]. However, increasing prevalence, early reoccurrence, and high mortality highlights it among the other stroke subtypes [2,3,4].

Most of cardioembolic strokes are associated with non-valvular atrial fibrillation (NVAF). [3]. The prevalence of NVAF is age dependent, as below the age of 55 it is less than 0.1%, reaching 9% by the age of 80 [5]. Because of ageing population, a 2.5-fold increase in patients with atrial fibrillation (AF) is expected in the next 50 years [5,6,7]. This population also has an increased risk of ischemic heart disease and congenital heart failure, which are considered high-risk sources of cardioembolism [1,4]. Although there are increasingly better treatment options for arterial hypertension, atherosclerosis, and dyslipidemia, that could reduce the prevalence of other stroke subtypes the incidence of cardioembolic stroke is expected to increase [2].

It is considered that the use of oral anticoagulants reduces stroke risk by up to 70% in patients with NVAF [8]. However, as described in the available literature, they are frequently underused, especially in elderly patients and patients with paroxysmal AF [9]. According to current American Heart Association guidelines, in elderly patients, the inadequate anticoagulation or no therapy at all is associated with higher bleeding risks, recurrent falls, and dementia [9]. The guidelines also suggest that AF patients who are not able to use any oral anticoagulants should receive oral aspirin or aspirin in combination with clopidogrel [9,10]. However, there is no evidence of significantly better stroke outcome in patients with AF taking antiplatelet agents versus not taking any antithrombotic medication [11].

In our paper we analyzed the long-term outcome of severely disabled (Modified Rankins Scale 4–5) cardioembolic stroke survivors depending on the prescribed antithrombotic secondary prevention medication.

## 2. Materials and Methods

In our study we retrospectively collected data for IS patients treated in P. Stradins Clinical University hospital, Riga, Latvia, from 2014 until 2017. Patients’ clinical data were collected using local stroke registry including patient’s demographic data, vascular risk factors, clinical findings, and laboratory results. Patients’ pre-event stroke risk was assessed by CHA_2_DS_2_-VASc score divided into low (0–1 points), moderate (2–3 points), and high risk (≥4 points) [12]. Bleeding risk was measured by HAS-BLED score, patients were divided in two groups: low risk of bleeding (0–3 points) and high risk of bleeding (≥4 points), and both scores were calculated retrospectively from available data [12]. The Latvian version of National Institutes of Health Stroke Scale (NIHSS-LV) and modified Rankin’s scale (mRS) were used to evaluate patients’ neurological and functional outcome at discharge [13]. Trial of ORG 10172 in acute stroke treatment (TOAST) classification was used to determine stroke etiology [1]. Only cardioembolic stroke patients diagnosed with NVAF and a severe neurological deficit (mRS 4–5) on discharge were included in the study. We excluded hemorrhagic stroke and other IS subtypes according to TOAST criteria (large-artery atherosclerosis, small-vessel occlusion, stroke of other determined etiology, and stroke of undetermined etiology). Patients with other possible high risk source of cardioembolism as mechanical prosthetic valve, mitral stenosis with AF, left atrial/atrial appendage thrombus, sick sinus syndrome, recent myocardial infarction (<4 weeks), left ventricular thrombus, dilated cardiomyopathy, akinetic left ventricular segment, atrial myxoma, and infective endocarditis were excluded [1]. Access to patients’ contact information and patients’ or their relatives’ consent to participate in the study was required to perform the follow-up.

Severely disabled stroke survivors were followed up by phone at 30/90/180/365 days after discharge. Patients’ functional outcomes were assessed using the adapted version of The Rankin Focused Assessment–Ambulation (RFA–A) [14]. Standardized questions were asked about patients’ usage of prescribed medication. Patients were divided in 4 groups according to prescribed secondary prevention medication (no medication, antiplatelet agents, vitamin K antagonists, and direct oral anticoagulants).

Collected data were analyzed with Microsoft® Office Excel (2016) and IBM SPSS Statistics 24. Descriptive statistics were used for continuous variables (SD, mean, median, quartiles, min, max) and categorical variables (number and percentage). For survival analysis, the Kaplan–Meier method and Cox proportional hazards ratio were applied to compare the results in all patient groups. Repeated measures mixed models were used of assessment of function outcome over time. The results were compared in four groups accordingly.

The study’s ethical aspects were evaluated and approved by The Ethics Committee of Riga Stradins University (RSU) (ethical committee approval, nr. 23, 29 March 2018). A verbal consent of participation was asked when contacting patients of their relatives by phone.

## 3. Results

### 3.1. Patient Characteristics

In the timespan of the study, a total of 4101 IS patients were treated in Pauls Stradins Clinical University Hospital, Department of Neurology, Riga, Latvia. Out of 1955 (47.7%) cases the stroke subtype was diagnosed as cardioembolic. Intrahospital mortality of cardioembolic stroke was 14.12%. At discharge, 746 (38.16%) of all cardioembolic stroke patients had unsatisfactory functional outcome of mRS 4–5 [15]. A total of 682 (91.42%) patients were followed up and included in data analysis.

The median age of patients was 80 (IQR = 75–85) years. Of these patients, 231 (31%) were males and 515 (69%) were females. We observed a difference in average age in different patient groups. The average age of patients receiving no antithrombotic treatment was 83 (IQR = 74–86) years and patients receiving antiplatelet agents 83 (IQR = 77–87) years, opposed to the average age of patients receiving Vitamin K antagonists (VKA), 78 (IQR = 72–84) years and 78 (IQR = 72–84) years of patients on direct oral anticoagulants (DOACs). This difference was statistically significant *p* < 0.005.

Most common risk factors were hypertension 606 (88.9%), dyslipidemia 524 (76.8%), congestive heart failure 422 (61.9%), and age over 70 years 602 (88.3%).

Patients with CHA2DS2-VASc severe score (>3 points) were 557 (81.7%). A total of 600 (88%) patients presented with low bleeding risk and HAS-BLED score between 0–3 points and 82 (12%) with high bleeding risk (HAS-BLED > 3 points).

The median total LV-NIHSS was 14 points (IQR = 8–18) on admission, and 10 points (IQR = 5–16) at discharge. The median LV-NIHSS on admission as well as on discharge was higher in patients receiving no antithrombotic medication and patients receiving antiplatelet agents. This difference was statistically significant *p* < 0.005.

On discharge, 51 (7.5%) patients were prescribed no antithrombotic therapy, 313 (45.9%) patients were discharged on antiplatelet agents, and 79 (11.6%) on VKA and 234 (35%) on DOACs. The patients’ characteristics in all treatment groups are seen in Table 1.

### 3.2. Survival Analysis

**Figure 1:**
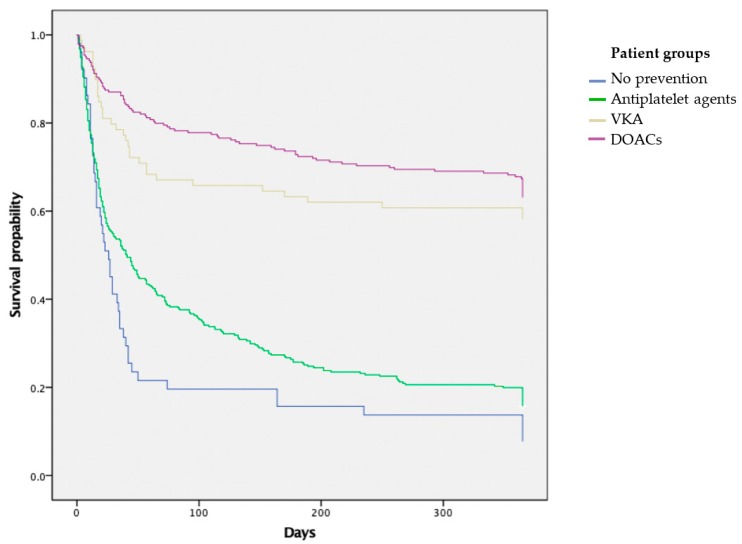
Cumulative survival in different treatment groups.

The crude probability of survival in the 682 IS patients is shown in Figure 1. The combined mortality for all treatment groups was 15.1% in 30 days, 23.8% in 90 days, 31% in 180 days and 36.8% in one year. However, these outcomes were relatively diverse among the treatment groups. In the first month after discharge, the crude probability of survival was lowest in patients that did not receive any prevention medication 63% (IQR = 48–78) and patients taking antiplatelet agents 75% (IQR = 64–86). The survival was significantly higher in patients taking VKA 87% (IQR = 81–93) and DOACs 92% (IQR = 88–95). In 90d after discharge, the crude probable survival rate in patients without secondary preventive medication was 56% (IQR = 33–76), in patients on antiplatelet agents 65% (IQR = 50–83), VKA 81% (IQR = 72–90) and 88% (IQRS = 82–94) in patients taking DOACs.

In 180 days, the crude probability of survival in patients not taking any secondary preventive medication was 53% (IQR = 31–76), 59% (IQR = 41–79) in patients taking antiplatelet agents, 79% (IQR = 68–89) in patients on VKA, and 84% (IQR = 76–92) in patients on DOACs. One-year crude probability of survival of patients not taking any preventive medication was 53% (IQR = 29–76), in patients taking antiplatelet agents it was 57% (IQR = 37–78), 78% (IQR = 68–88) of patients on VKA, and 81% (IQR = 72–90) in patients on DOACs.

Patients in no-prophylaxis and antiplatelet agent groups were statistically significantly older and had a higher LV-NIHSS at baseline. To compare all treatment groups, cox regression analyses was performed, including the patient age and baseline LV-NIHSS as possible confounders.

No difference was observed when comparing no-prophylaxis group and patients taking antiplatelet agents, with hazards ratio (HR) of 0.778 (*p* = 0.119). The HR between patients in no-prophylaxis group and patients taking VKA was 0.313 (*p* < 0.001) and patients on DOACs 0.246 (*p* < 0.001), which was statistically significant.

When comparing antiplatelet group with patients taking VKA, the HR was 2.485, which, based on confidence interval (CI), was statistically significant. The HR between antiplatelet group and patients taking DOACs was 3.162 and was also statistically significant.

When comparing VKA and patients taking DOACs with HR of 1.272, according to the CI it was not statistically significant.

After the adjustment for confounders, the secondary prevention showed a significant statistical importance, with patients taking VKA or DOACs having statistically significantly less hazards then patients on no prophylaxis of antiplatelet agents.

### 3.3. Functional Outcome Analysis

**Figure 2:**
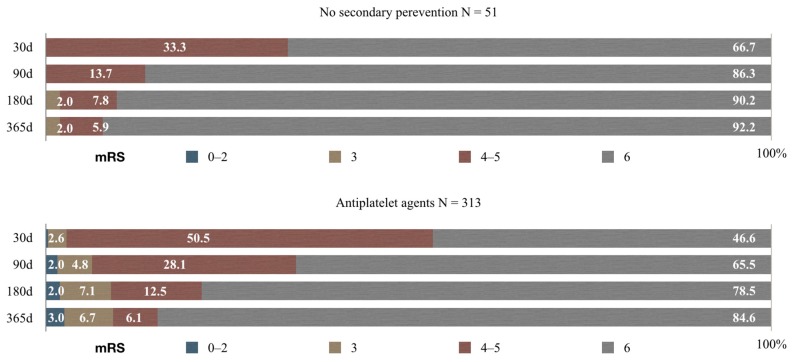
Functional outcome in patients without prevention medication and in patients on antiplatelet agents.

Function outcomes overtime in patients without antithrombotic treatment and in patients taking antiplatelet agents are shown in Figure 2.

In 30 days since discharge of 51 patients not taking any prevention medication, 17 (33.3%) were severely disabled and 34 (66.7%) had died. In 90 days, 7 (13.7%) had an mRS of 4–5 and 44 (86.3%) had died. In 180 days, 1 (1.9%) patient had a moderate neurological deficit, 4 (7.8%) were severely disabled, and 46 (90.3%) patients had died. One year after discharge, 1 (1.9%) patient had a moderate neurological deficit, 3 (5.9%) were severely disabled, and 47 (92.2%) patients had died.

In patients taking antiplatelet agents, after 30 d 1 (0.3%) patient had satisfactory functional outcome (mRS 0–2), 8 (2.5%) had a moderate deficit, 158 (50.5%) were severely disabled, and 146 (46.7%) had died. In 90d after discharge, 5 (1.6%) patients had mRS 0–2, 15 (4.8%) mRS 3, 88 (28.1%) were still severely disabled, and 205 (65.5%) had died. In 180 d after discharge 6 (1.9%) had a favorable outcome, 22 (7.0%) had a moderate deficit, 39 (12.5%) an mRS 4–5 and 245 (78.6%) had died. One year after discharge 8 (2.6%) had mRS 0–2, 21 (6.7%), 19 (6.1%) an mRS 4–5 and 264 (84.6%) had died.

**Figure 3:**
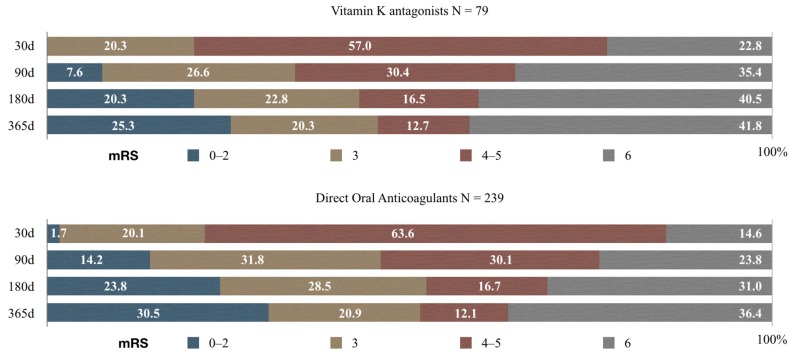
Functional outcome in patients on VKA and in patients on DOACs.

Function outcomes overtime in patients taking VKA and in patients on DOACs are shown in Figure 3.

In patients on VKA, 30 d after discharge 16 (20.3%) had a moderate deficit, 45 (56.7%) were severely disabled, and 18 (33%) had died. In 90 d after discharge, 6 (7.6%) patients had mRS 0–2, 21 (26.6%) mRS 3, 13 (16.5%) were still severely disabled, and 28 (49.3%) had died. In 180 d after discharge, 16 (20.2%) had a favorable outcome, 18 (22.8%) had a moderate deficit, 13 (16.5%) an mRS 4–5, and 32 (40.5%) had died. One year after discharge, 20 (25.3%) had mRS 0–2, 16 (20.3%), 10 (12.6%) an mRS 4–5, and 33 (41.8%) had died.

In patients taking DOACs, 30 d after discharge, 4 (1.7%) had a light neurological deficit, 48 (20.1%) had a moderate deficit, 152 (63.6%) were severely disabled, and 35 (14.6%) patients had died. In 90 d after discharge, 34 (14.2%) patients had mRS 0–2, 76 (31.8%) mRS 3, 72 (30.1%) were severely disabled, and 57 (23.9%) had died. In 180 d after discharge, 57 (23.8%) had a favorable outcome, 68 (28.5%) had a moderate deficit, 30 (16.7%) an mRS 4–5, and 74 (31%) had died. One year after discharge, 73 (31%) had mRS 0–2, 50 (20.9%), 29 (12.1%) were still severely disabled, and 87 (36.4%) had died.

## 4. Discussion

Our study looked into the long-term functional outcome of cardioembolic IS survivors with severe neurological deficit diagnosed with NVAF, analyzing the main risk factors in four groups of patients according to medication used for secondary stroke prophylaxis. Cardioembolic strokes made a 47.7% of all stroke cases in our clinic in the timespan of the study, which is a high proportion comparing with world data [16]. Increase in frequency of cardioembolic strokes is a tendency worldwide and this highlights the importance of changes and impact what anticoagulation can have [17]. This situation in Latvia has been studied before regarding high prevalence of cardioembolic IS patients. In our study population, we found that 38.16% of all cardioembolic stroke patients had an unfavorable functional outcome of mRS 4–5 at discharge. In similar studies, this rate has been as high as 57.4% [18]. As previously described in study by Jurjans, et al. (2015), it is proved that primary cardioembolic stroke prevention in Latvia is insufficient and mortality rate is significantly higher in patients that use no secondary prevention medication or antiplatelet agents compared to the patients that use oral anticoagulants [19].

According to Global burden of disease estimates, the annual number of incidence IS in Latvia is 10,282 [20]. There are currently 7 operating stroke units in Latvia with 3.6 stoke units per million inhabitants with 30% of patients receiving stroke unit care [21]. According to Statistical Yearbook of Healthcare in Latvia 2017, the death rate of IS has a tendency to decrease and reaches 80.6 per 100,000 inhabitants in 2017.

Following recent IS in patients with AF, one third of patients are not managed according to current guidelines for preventive medication [22]. For patients with severe stroke (mRS 4–5), there are no current clinical trials or guideline recommendations available. Nonadherence with current recommendations for Oral anticoagulants (OAC) alone in patients with IS and AF may occur for several reasons. McGrath et al. (2014) state that, firstly, clinicians may believe that their individual patients are not represented in the evidence-base that supports current guidelines recommendations, because they present a higher risk of thromboembolism or a lower risk of major bleeding than the average patient included in clinical trials. Secondly, in patients with severe stroke, withholding of any antithrombotic therapy may be part of an overall palliative approach to care [22].

Numerous studies have described the elevated risk of IS in patients with AF and the increasing severity of AF–associated strokes and 30-day mortality [23,24,25]. Fang et al. (2012) assessed the impact of anticoagulation on 30-day mortality from IS versus ICH in a large community-based cohort of 1025 IS patients with AF. Compared with no antithrombotic therapy, warfarin was associated with reduced Rankin score and lower 30-day mortality from IS (adjusted OR, 0.64; 95% CI, 0.45–0.91) [25]. Emer R. McGrath et al. (2014): comparing OAC alone on discharge, no antithrombotic therapy (HR, 1.57; 95% CI, 1.26–1.95) and antiplatelet therapy alone (HR, 1.42; 95% CI, 1.23–1.64) were associated with an increased risk of death over 4 years, whereas combination OAC and antiplatelet therapy was associated with a similar risk of death (HR, 0.94; 95% CI, 0.82–1.08) [22]. Aruz A. et al. (2017) had study with 129 consecutive acute IS NVAF patients (mean age 70.2 ± 10.1 years). After a median follow-up of 17 months (IQR 6–54.5), 35.6% patients had bad outcome, and 36.4% died [26]. Accurate estimates of IS patient long-term survival are needed to make a decision whether to use higher risk therapies, such as anticoagulants, to prevent a stroke [27].

Our selected risk factors are associated with poor functional outcome. Possible factors that have been described for high mortality in NVAF include age, hypertension, diabetes mellitus, severe initial stroke symptoms, dyslipidemia, ischemic heart disease, congenital heart disease [23,28,29,30]. Risk is estimated using CHA2DS2-VASc and HAS-BLED scores.

In our study, long-term survival after a cardioembolic IS strongly correlated with the severity of the stroke at discharge (LV-NIHSS), and the patients’ ages. As the functional deficits and patients age increased, the mortality rates also increased significantly. Analyzing LV-NIHSS score, VKA (N = 79) group had lowest median score of LV-NIHSS on discharge—6 (IQR = 6–10). Better clinical state for VKA group at discharge from hospital could be explained with a need for better cooperation for INR control. Other common risk factors had no strong impact, as independent factors for bad outcomes and mortality in our study. We did not find significantly high correlation among them.

Our study demonstrated a mortality progression over time. It was significantly lower in the novel oral anticoagulant-group (30-day mortality 15.1%; 1-year mortality 36.4%) than in the no medication-group (30-day mortality 34%; 1-year mortality 94.1%). These findings are similar to results from previous studies [23,28,30]. However, no other study has studied this specific group of cardioembolic IS survivors with severe neurological deficit diagnosed with NVAF in four groups regarding secondary preventive medication. Our study confirms that the impact of cardioembolic IS on survival extends far beyond the initial 30-day period. Better functional outcome is expected if patient is discharged from hospital with novel oral anticoagulant alone as secondary prevention medication. The poor survival that we observed after AF-associated stroke is considerably worse than the survival reported for patients after stroke from all causes together [31,32].

In a study by Appelros et al. (2003) 37% of survivors (N = 253) were functionally dependent (mRS ≥3) at 1 year after stroke [33]. In Sanfallt et al. (2019) study, the corresponding proportion was 45.3% [34]. In a Swedish study (N = 35 064) Ulberg T. et al. (2015), a decline in mobility was seen in 43% patients between discharge and 1-year follow-up [35]. An Auckland study from 2010 reported poor functional outcome (mRS ≥ 3) in 31.4% of survivors (N = 418) [36]. In these and other previous researches, it was not studied how secondary preventive medication affect long-term functional outcome in severe stroke patients with NVAF.

In our study during the course of follow-up (30 d, 90 d, 180 d, 365 d), proportion of functional dependency in survivors at each time point decreased and in all patient groups there were patients with improved functional status. In direct oral anticoagulant group, up to 31% of patients who were discharged from hospital with mRS 4–5 after 365 d follow-up showed significant improvement in functional outcome with mRS 0–2. The outcome was less favorable for patients taking no medication, as there were no patients with mRS 0–2 at 365 d follow-up and only 2.0% of these patients had mRS 3. This shows that even patients with unsatisfactory functional level (mRS, 4–5) at discharge can improve in a long-term to good functional outcome (mRS, 0–2) and that this is significantly affected by the use of secondary preventive medication.

The most common reason for severely disabled patients to die after a severe stroke are recurrent embolic events as IS, myocardial infarction, mesenteric thrombosis, pulmonary artery thromboembolism, and complications for patient being bedbound: decubitus, pneumonia, urinary tract infections etc. [37]. The usage of oral anticoagulants has proven to reduce all these types of embolic events, and this explains lower mortality rates in patients taking VKA or DOACs [27].

Strengths of our study include systematically collected data from all consecutive IS patients in single tertiary center during four-year period. Standard protocols and scales have been used (LV-NIHSS, mRS, CHA2DS2-VASc, HAS-BLED) with standardized follow-up procedure (RFA-A). In our region, this is the first long-term study in NVAF stroke survivors with unsatisfactory functional outcome of mRS 4–5. As there is not sufficient data for such patients, it is important to show long-term results considering secondary prevention medication.

Our study also has some limitations. The statistically significant difference in age and in baseline severity of stroke (LV-NIHSS on admission and at discharge) might lead to offset in the overall results. Also, the patient population treated with OAC therapy was limited by socioeconomic factors; antiplatelet therapy is generally seen as a low-cost alternative compared with direct oral anticoagulants. This factor could have an impact on the choice of antithrombotic medication.

## 5. Conclusions

Anticoagulant use in secondary prevention predicts a better functional outcome and higher survival rate in patients with severe cardioembolic stroke due to NVAF. Therefore, severe neurological deficit must not be a reason of restriction of anticoagulation.

The increased age and baseline severity of stroke (LV-NIHSS on admission and at discharge) might be considered to be the reason for withholding anticoagulation.

The use of antiplatelet agents showed only slight improvement in long-term survival rate and functional outcome as patients without any antithrombotic treatment, but this finding was not statistically significant. Therefore, antiplatelet therapy should not be recommended in this patient group.

Our study offers an answer to how to manage antithrombotic treatment in patients with severe cardioembolic stroke in the absence of guidelines for this patient group.

Research shows a necessity to improve secondary prevention of cardioembolic stroke in Latvia by increasing the frequency of anticoagulant usage.

## Figures and Tables

**Table 1:** Patients’ demographic characteristics.

	No Prophylaxis *N* = 51 (7.5%)	Antiplatelet Agents *N* = 313 (45.9%)	VKA *N* = 79 (11.6%)	DOACs *N* = 234 (35%)	Total *N* = 682 (%)	*p*
Average age (IQR ^1^)	83 (IQR = 74–86)	83 (IQR = 77–87)	78 (IQR = 72–84)	78 (IQR = 72–84)	80 (IQR = 75–85)	<0.005
≥70 years old	45 (88.2%)	295 (94.2%)	64 (81%)	198 (84.6%)	602 (88.3%)	<0.005
Hypertension	44 (86.3%)	287 (91.7%)	66 (83.5%)	209 (89.3%)	606 (88.9%)	0.131
Diabetes mellitus	4 (7.8%)	42 (13.1%)	12 (15.2%)	36 (15.4%)	94 (13.8%)	0.570
Dyslipidemia	38 (74.5%)	237 (75.7%)	61 (77.2%)	188 (80.3%)	524 (76.8%)	0.843
Ischemic heart disease	10 (19.6%)	52 (16.6%)	15 (19%)	47 (20.1%)	124 (18.2%)	0.807
Congenital heart disease	31 (60.8%)	205 (65.5%)	46 (58.2%)	140 (59.8%)	422 (61.9%)	0.346
History of stroke or TIA	21 (41.2%)	118 (37.7%)	30 (38%)	86 (36.8%)	255 (36.4%)	0.910
CHA_2_DS_2_-VASc score						
0–3 (low and moderate risk)	2 (3.9%)	5 (1.6%)	6 (7.6%)	9 (3.8%)	22 (3.2%)	0.51
>3 (severe risk)	49 (96.1%)	308 (98.4%)	73 (92.4%)	230 (96.2%)	660 (96.8%)	0.51
HAS-BLED score						
0–3 (low bleeding risk)	35 (68.6%)	202 (64.5%)	57 (72.2%)	173 (73.9%)	467 (68.5%)	0.218
>3 (high bleeding risk)	16 (31.4%)	111 (35.5%)	22 (27.8%)	66 (26.1%)	215 (31.5%)	0.218
LV-NIHSS on admission	15 (IQR = 11–20)	15 (IQR = 9–19)	10 (IQR = 6–15)	13 (IQR = 7–17)	14 (IQR = 8–18)	<0.005
LV-NIHSS on discharge	13 (IQR = 7–16)	12 (IQR = 6–17)	6 (IQR = 6–10)	8 (IQR = 4–14)	10 (IQR = 5–16)	<0.005

^1^ IQR—interquartile range.
